# Supplementary material for: Virus-modified paraspeckle-like condensates are hubs for viral RNA processing and their formation drives genomic instability
Source: Nat Commun. 2024 Nov 26;15:10240. doi: 10.1038/s41467-024-54592-5 (PMC11599752; doi:10.1038/s41467-024-54592-5)

Virus-modified paraspeckle-like condensates are hubs for viral RNA processing and their formation drives genomic instability

Katherine L Harper[1], Elena M Harrington, Connor Hayward, Chinedu A Anene, Wiyada Wongwiwat, Robert E White and Adrian Whitehouse

Supplementary Figures 1-7

Supplementary Table 1

Supplementary Source Data

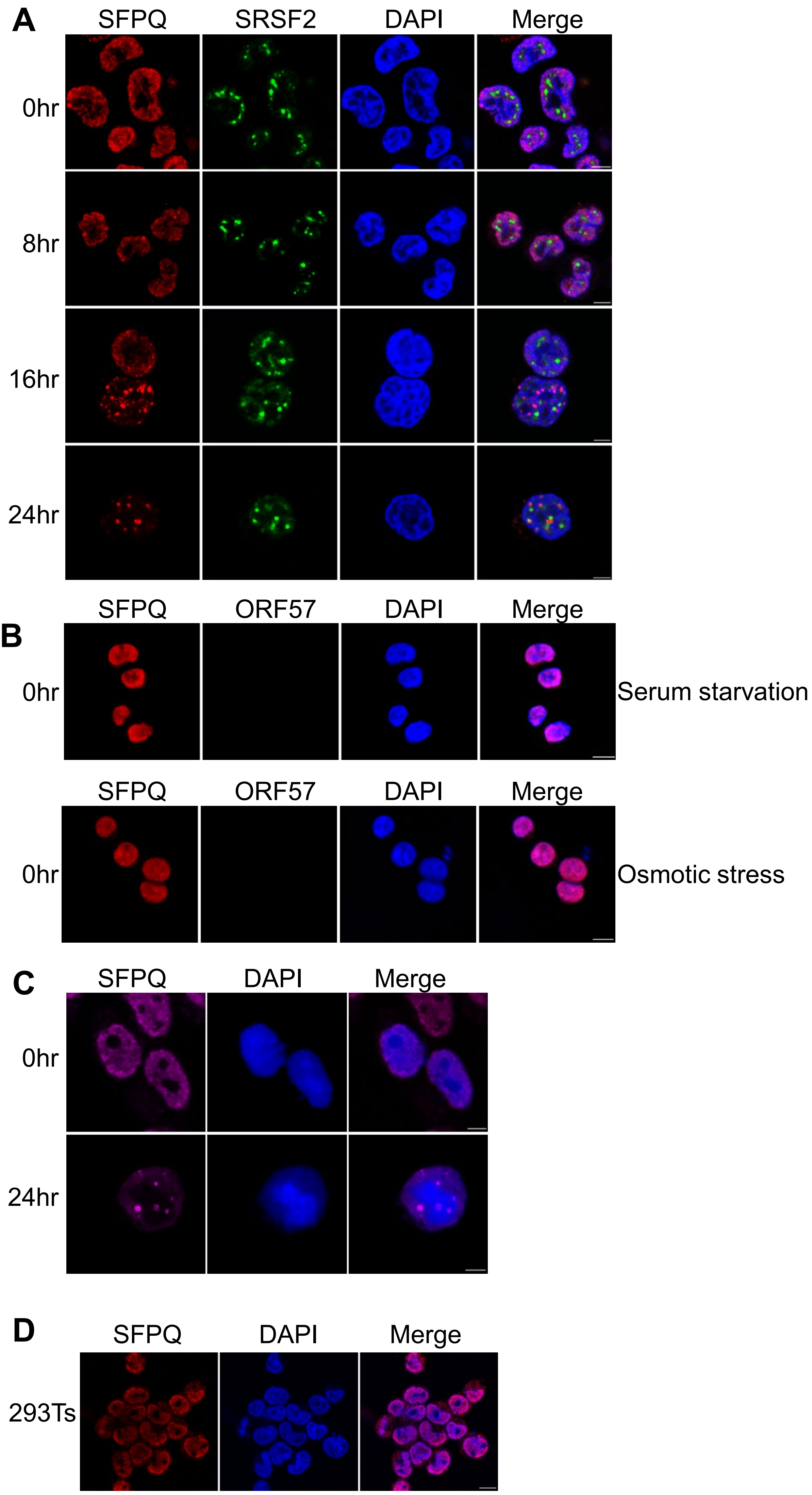

A
SFPQ
SRSF2
DAPI
Merge
0hr
8hr
16hr
24hr
B
SFPQ
ORF57
DAPI
Merge
0hr
Serum starvation
SFPQ
ORF57
DAPI
Merge
0hr
Osmotic stress
C
SFPQ
DAPI
Merge
0hr
24hr
D
SFPQ
DAPI
Merge
293Ts

Figure S1 (A-D)

Figure S1:

(A). IF of TREx-BCBL1-RTA cells at 0, 8, 16 and 24 hours staining for SFPQ (red), SRSF2 (green) and DAPI (blue).

(**B).** IF of TREx-BCBL1-RTA cells during latency, with a 24 hour pre-treatment serum starvation (top row) or exposed to osmotic stress (bottom row). Stained for SFPQ (red), ORF57 (green) and DAPI (blue).

(C). IF of HEK-293T-rKSHV.219 cells at 0 and 24 hours post-reactivation with staining against SFPQ (purple) and DAPI (blue).

(D). IF of HEK-293T cells with staining against SFPQ (red) and DAPI (blue).

Scale bars are 5 μm in A,C and 10 μm in B and D.

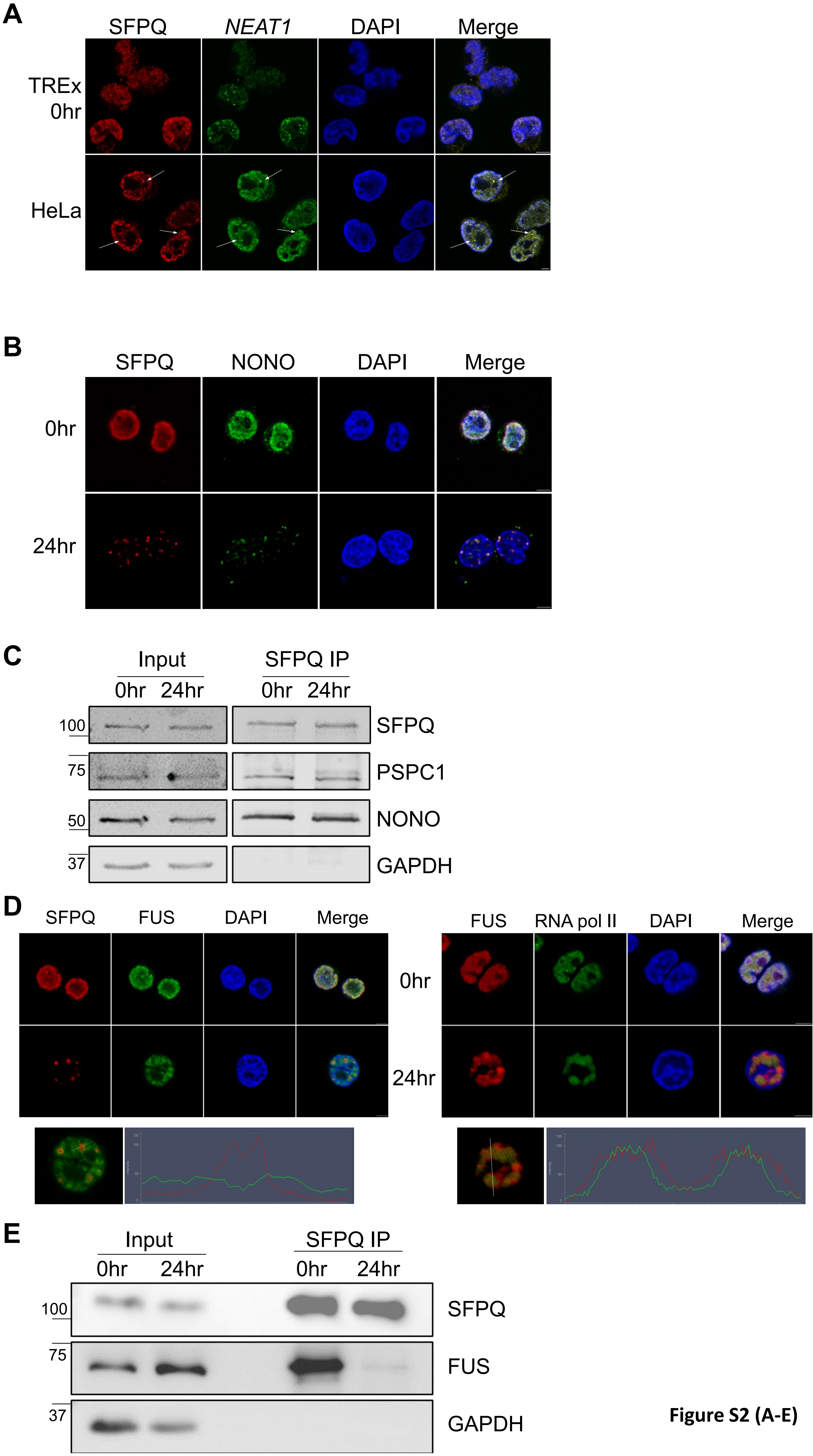

A
SFPQ
NEAT1
DAPI
Merge
TREx 0hr
HeLa
B
SFPQ
NONO
DAPI
Merge
0hr
24hr
C
Input
SFPQ IP
0hr
24hr
0hr
24hr
100
75
50
37
SFPQ
PSPC1
NONO
GAPDH
D
SFPQ
FUS
DAPI
Merge
0hr
24hr
FUS
RNA pol II
DAPI
Merge
E
Input
SFPQ IP
0hr
24hr
0hr
24hr
100
75
37
SFPQ
FUS
GAPDH

Figure S2 (A-E)

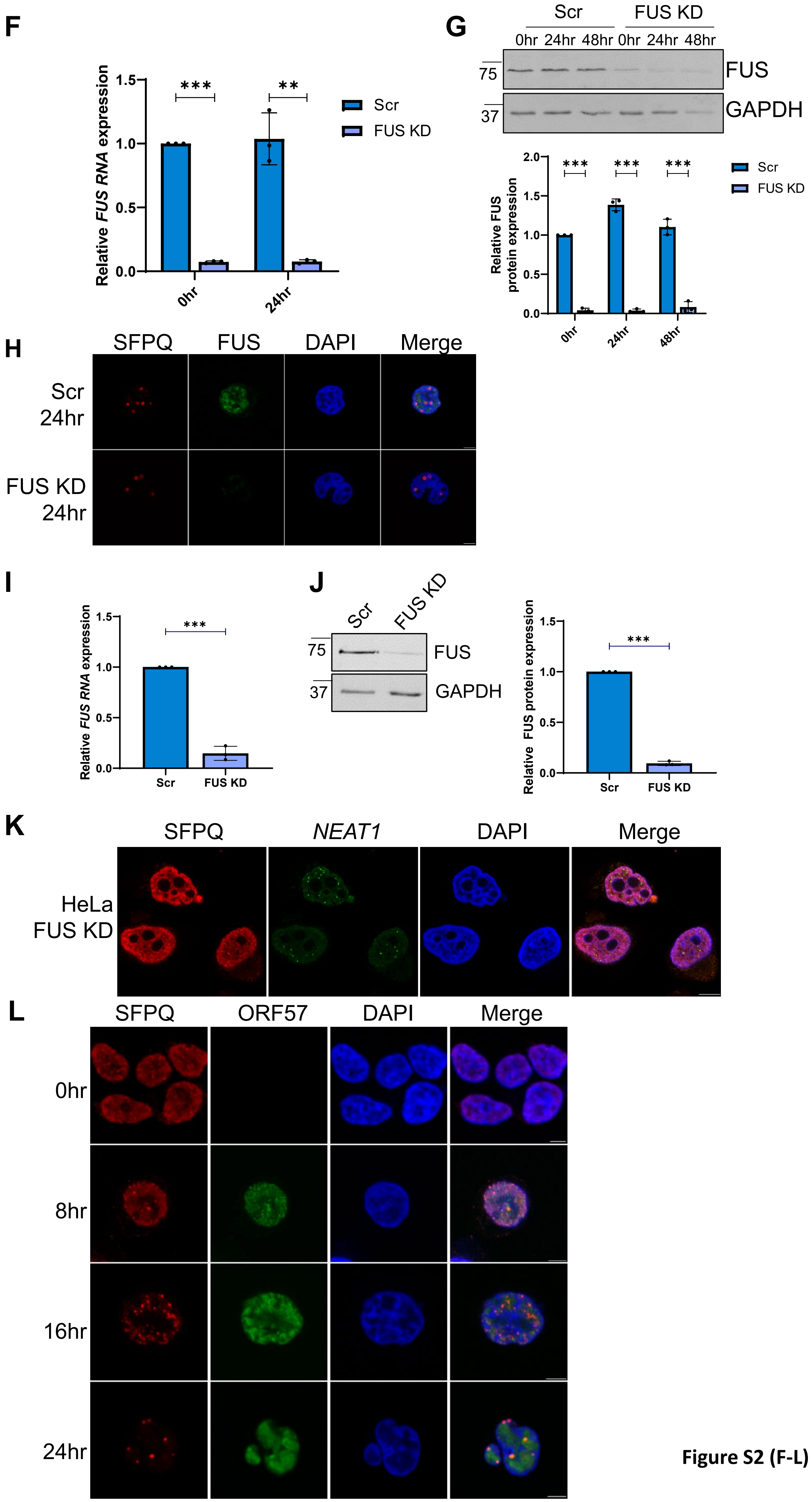

F
Relative FUS RNA expression
1.5
1.0
0.5
0.0
***
**
0hr
24hr
Scr
FUS KD
G
Scr
FUS KD
0hr 24hr 48hr 0hr 24hr 48hr
75
37
FUS
GAPDH
Relative FUS protein expression
2.0
1.5
1.0
0.5
0.0
***
***
***
0hr
24hr
48hr
Scr
FUS KD
H
SFPQ
FUS
DAPI
Merge
Scr 24hr
FUS KD 24hr
I
Relative FUS RNA expression
1.5
1.0
0.5
0.0
***
Scr
FUS KD
J
Scr
FUS KD
75
37
FUS
GAPDH
Relative FUS protein expression
1.5
1.0
0.5
0.0
***
Scr
FUS KD
K
SFPQ
NEAT1
DAPI
Merge
HeLa FUS KD
L
SFPQ
ORF57
DAPI
Merge
0hr
8hr
16hr
24hr

Figure S2 (F-L)

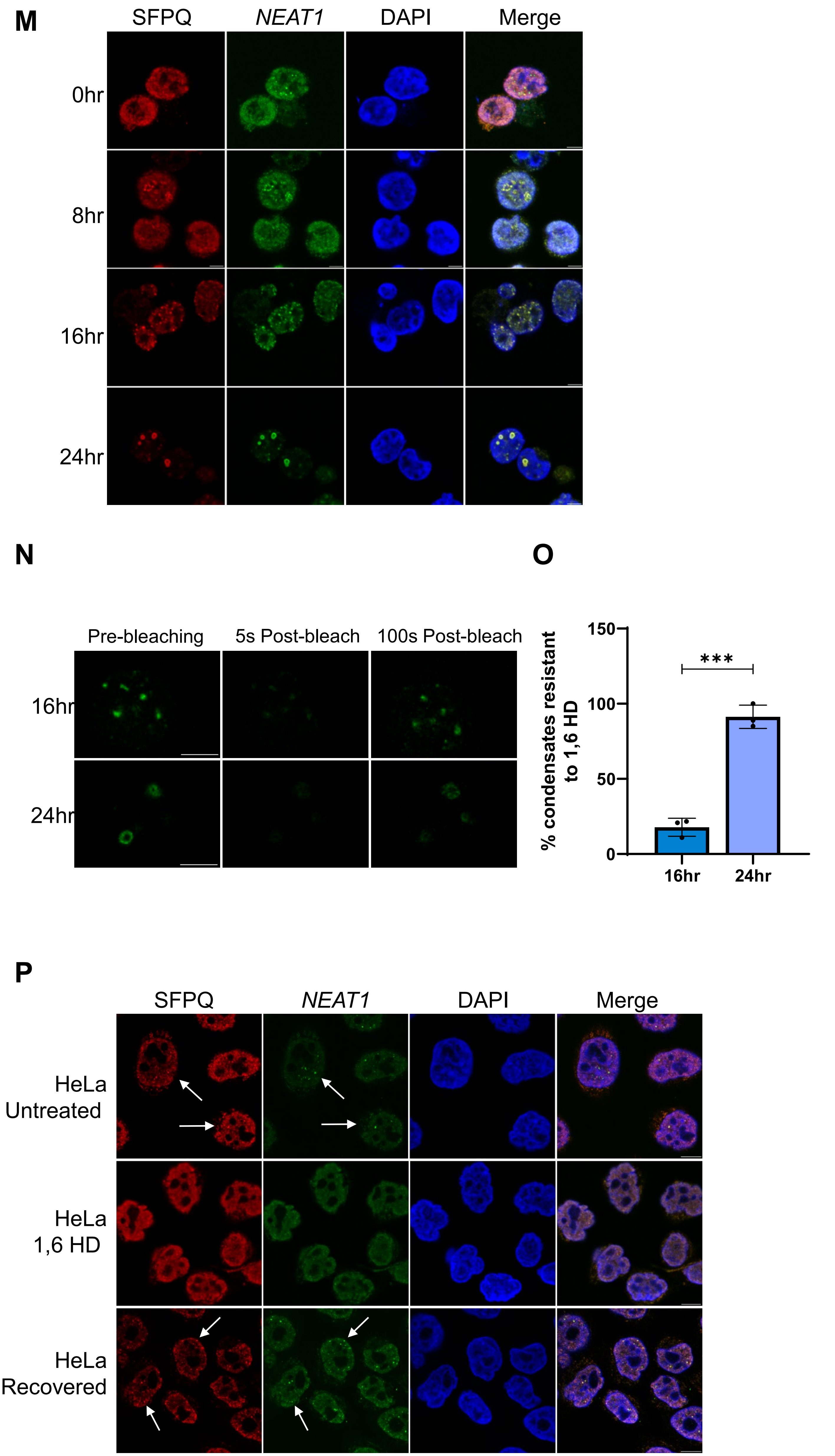
M
SFPQ
NEAT1
DAPI
Merge
0hr
8hr
16hr
24hr
N
Pre-bleaching
5s Post-bleach
100s Post-bleach
16hr
24hr
O
% condensates resistant to 1,6 HD
150
100
50
0
***
16hr
24hr
P
SFPQ
NEAT1
DAPI
Merge
HeLa Untreated
HeLa 1,6 HD
HeLa Recovered

Figure S2 (M-P)

Figure S2:

(A). IF of latent TREx or HeLa cells with staining for SFPQ (red), *NEAT1* (green) and DAPI (blue). Arrows highlight the canonical paraspeckles.

(B). IF of TREx at 0 and 24 hours with staining for SFPQ (red), NONO (green) and DAPI (blue).

(C). Representative blot of co-IP of SFPQ probed with antibodies against SFPQ, PSPC1, NONO and GAPDH (n=3).

(D). IF of TREx at 0 and 24 hours with staining for SFPQ (red), FUS (green) and DAPI (blue) with intensity analysis performed by Zen Blue (left). IF of TREx at 0 and 24 hours with staining for FUS (red), RNA pol II (green) and DAPI (blue) with intensity analysis performed by Zen Blue (right).

(E). Representative composite western blot of SFPQ co-IP in TREx at 0 and 24 hours with antibodies against SFPQ, FUS and GAPDH. n=3.

(F). qPCR of *FUS* in scr and FUS KD TREx cells at 0 and 24 hours. GAPDH was used a housekeeper, n=3. P values are <0.0001 and 0.0012.

(G). Representative western blot for levels of FUS in scr and FUS KD TREx cells at 0, 24 and 48 hours post-lytic induction. GAPDH was used as a loading control, densitometry analysis was performed on n=3. P values are <0.0001, <0.0001 and 0.0001.

(H). IF of scrambled and FUS KD TREx cells at 24 hours with staining for SFPQ (red), FUS (green) and DAPI (blue).

(I). qPCR of *FUS* in scr and FUS KD HeLa cells. P value is <0.0001.

(J). Representative western blot for levels of FUS in scr and FUS KD Hela cells. GAPDH was used as a loading control, densitometry analysis was performed on n=3. P value is <0.0001.

(K). IF of FUS KD HeLa cells with staining for SFPQ (red), *NEAT1* (green) and DAPI (blue).

(L). IF of TREx cells at 0, 8, 16 and 24 hours with SFPQ (red), DAPI (blue) staining and ORF57 (green).

(M). IF of TREx cells at 0, 8, 16 and 24 hours with SFPQ (red), DAPI (blue) staining and *NEAT1* (green).

(N). FRAP images of GFP-SFPQ O/E TREx cells at 16 and 24 hours post-reactivation. Images were taken 5 seconds pre-bleaching, 5 seconds post-bleaching and 100 seconds post-bleaching.

(O). Percentage of reactivated cells with v-mPS resistant to 1,6-hexanediol treatment at 16 and 24 hours post reactivation. For each biological repeat, 20 cells were counted. N=3. P value is 0.0002.

(P). IF of HeLa cells stained for SFPQ (red), *NEAT1* (green) and DAPI (blue). Cells were untreated (row 1), 1,6 HD treated (row 2), or 1,6 HD treated and allowed to recover for 30 minutes (row 3). White arrows are used to highlight paraspeckles in untreated and recovered cells.

Scale bars are 5 µm except in K, P (10 µm),. All repeats are biological. In F-J, O data are presented as mean ± SD. Unpaired two-tailed student T-test. *P < 0.05, **P < 0.01 and ***P < 0.001

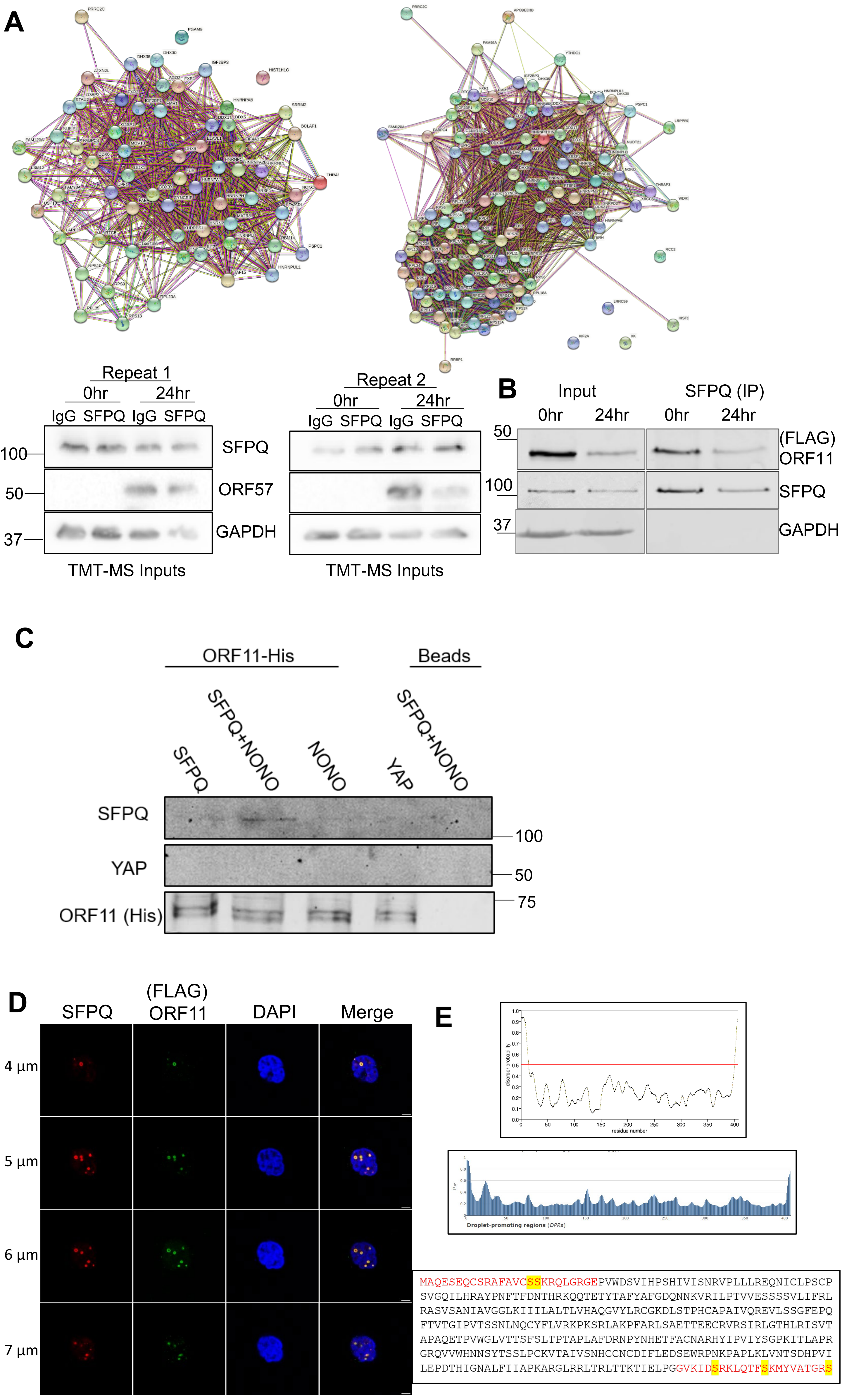
A
Repeat 1
0hr
24hr
IgG SFPQ IgG SFPQ
SFPQ
ORF57
GAPDH
100
50
37
TMT-MS Inputs
Repeat 2
0hr
24hr
IgG SFPQ IgG SFPQ
TMT-MS Inputs
B
Input
SFPQ (IP)
0hr 24hr 0hr 24hr
50
100
37
(FLAG)
ORF11
SFPQ
GAPDH
C
ORF11-His
Beads
SFPQ
SFPQ+NONO
NONO
YAP
SFPQ+NONO
SFPQ
YAP
ORF11 (His)
100
50
75
D
SFPQ
(FLAG)
ORF11
DAPI
Merge
4 µm
5 µm
6 µm
7 µm
E
disorder probability
residue number
Droplet-promoting regions (DPRs)
MAQESEQCSRAFAVCSSKRQLGRGEPVWDSVIHPSHIVISNRVPLLLREQNICLPSCP
SVGQILHRAYPNFTFDNTHRKQQTETYTAFYAFGDQNNKVRILPTVVESSSSVLIFRL
RASVSANIAVGGLKIIILALTLVHAQGVYLRCGKDLSTPHCAPAIVQREVLSSGFEPQ
FTVTGIPVTSSNLNQCYFLVRKPKSRLAKPFARLSAETTEECRVRSIRLGTHLRISVT
APAQETPVWGLVTTSFSLTPTAPLAFDRNPYNHETFACNARHYIPVIYSGPKITLAPR
GRQVVWHNNSYTSSLPCKVTAIVSNHCCNCDIFLEDSEWRPNKPAPLKLVNTSDHPVI
LEPDTHIGNALFIIAPKARGLRRLTRLTTKTIELPGGVKIDSRKLQTFSKMYVATGRS

Figure S3(A-E)

F

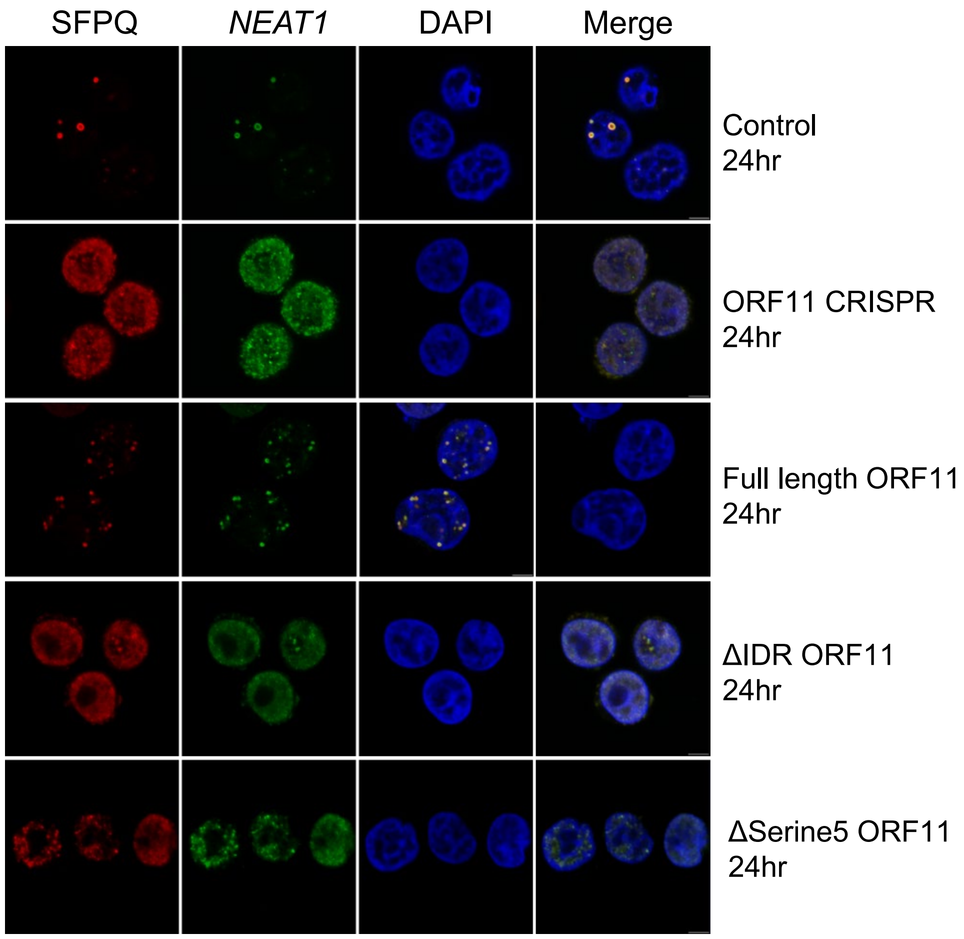

G

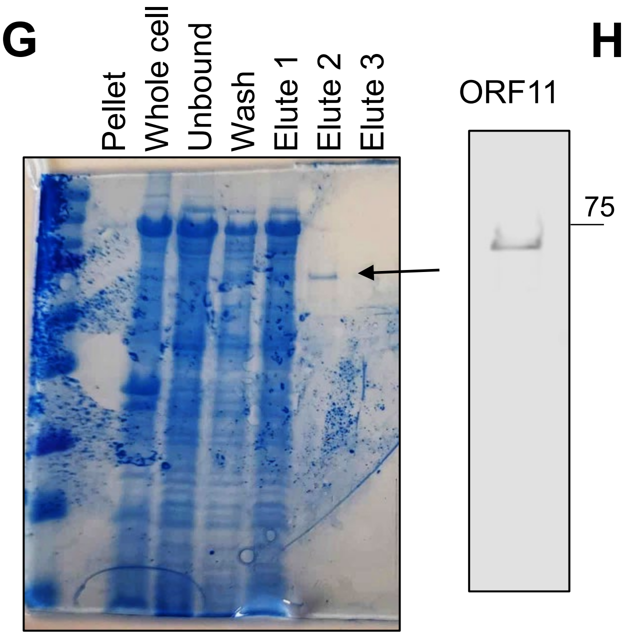

H

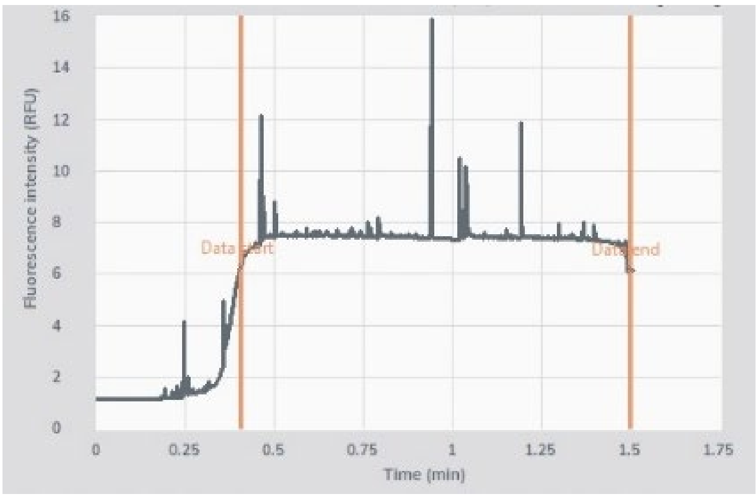

I

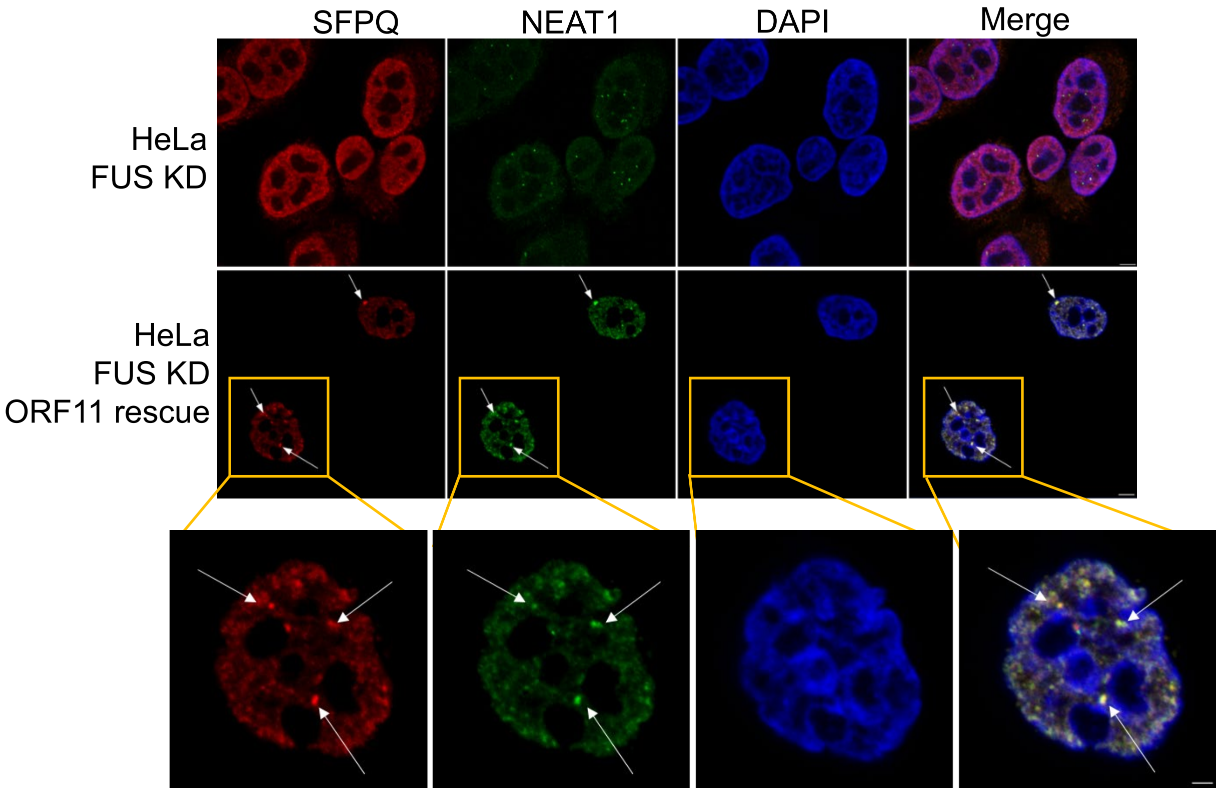

Figure S3(F-I)

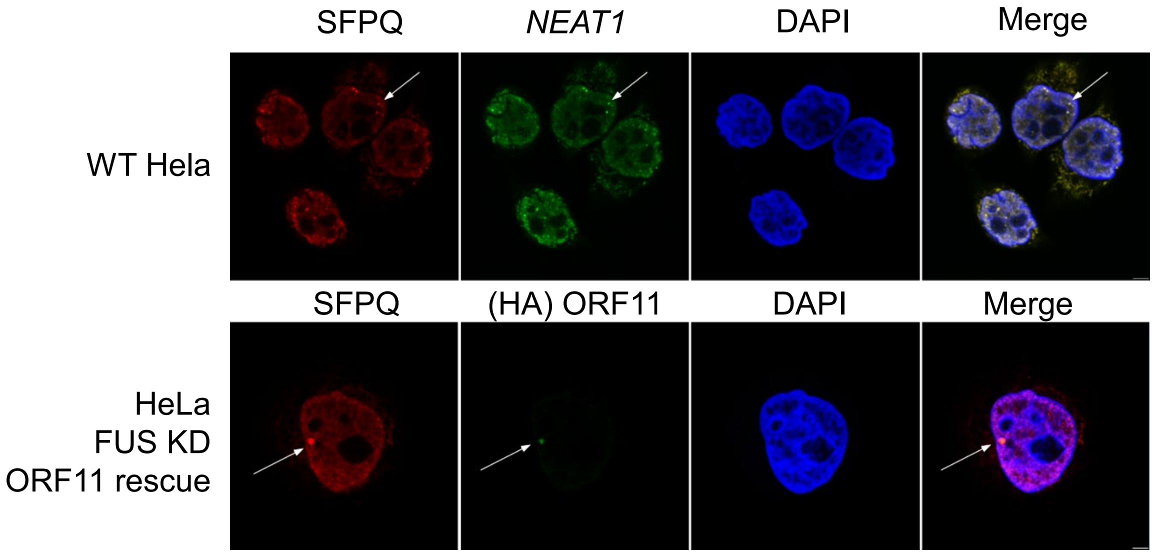

J

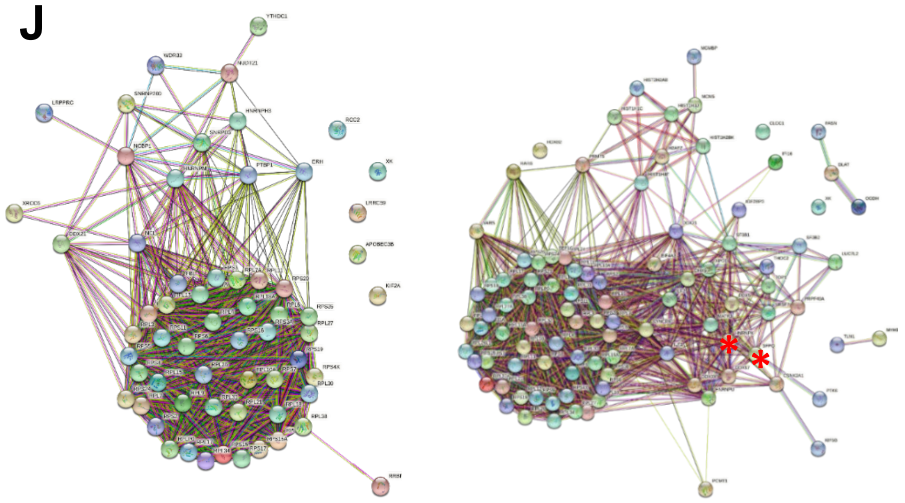

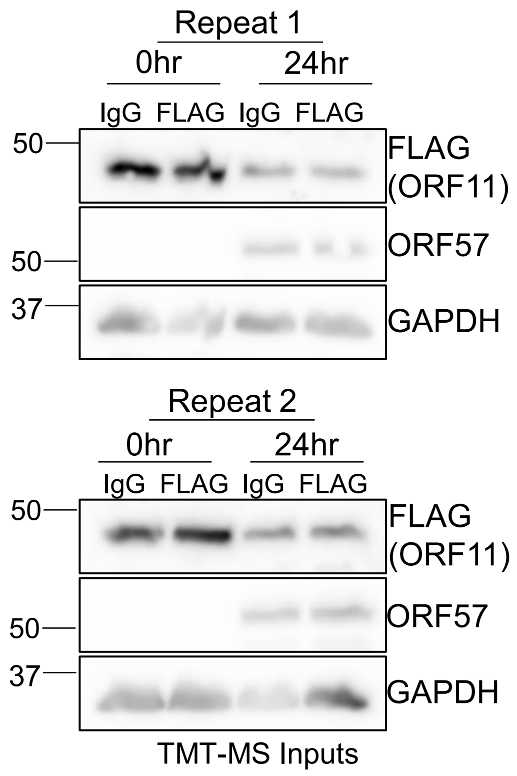

K

DEAD/DEAH box helicases	hnRNPs	Other RNA processing factors
DDX1	hnRNP A1	ADAR
DDX17	hnRNP A2B1	APOBEC3B
DDX21	hnRNP AB	ELAV1
DDX3X	hnRNP C	ERH
DDX5	hnRNP D	FAM98A
DHX30	hnRNP DL	FBL
DHX36	hnRNP H1	FMR1
DHX9	hnRNP H3	IGF2BP3
	hnRNP K	LUC7L2
	hnRNP L	MOV10
	hnRNP M	NCL
	hnRNP Q	PABPC1
	hnRNP R	PABPC4
	hnRNP U	PRPF40A
	hnRNP UL1	PTBP1
		RARS1
		RTCB
		RTRAF
		SF3BP
		SNRNP200
		SNRPA
		SNRPD3
		SRSF1
		UPF1
		VARS1
		WDR33
		YTHDC1

Figure S3(I-K)

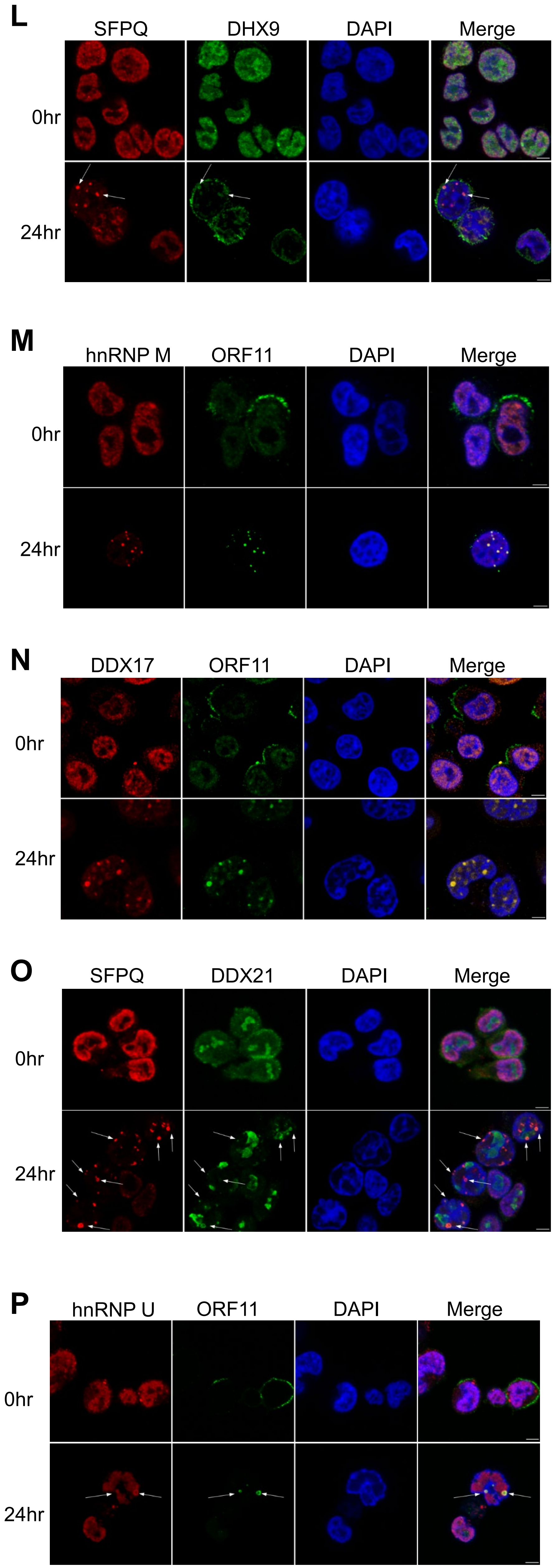
L
SFPQ
DHX9
DAPI
Merge
0hr
24hr
M
hnRNP M
ORF11
DAPI
Merge
0hr
24hr
N
DDX17
ORF11
DAPI
Merge
0hr
24hr
O
SFPQ
DDX21
DAPI
Merge
0hr
24hr
P
hnRNP U
ORF11
DAPI
Merge
0hr
24hr

Figure S3(L-P)

Figure S3:

(A). String analysis of TMT LC-MS/MS of SFPQ Co-IPs in TREx-BCBL1-RTA cells at 0 (left) and 24 hours (right). (n=2). Inputs of the cell lysates used for the Co-IPs were taken and analysed via western blot to confirm presence of SFPQ and lytic replication via ORF57 expression.

(B). Representative western blot for SFPQ Co-IPs in FLAG-ORF11-TREx-BCBL1-RTA cells at 0 and 24 hours. Membranes were probed for SFPQ, GAPDH and FLAG (ORF11). N=3.

(C). Representative western blot for *in vitro* ORF11 pulldowns. Purified SFPQ, SFPQ and NONO, NONO and YAP was washed over unbound or ORF11 bound beads. Membranes were probed for SFPQ, YAP and ORF11 (his). N=2.

(D). IF analysis taken through different z-planes of a cell stained for SFPQ (red) FLAG/ORF11 (green) and DAPI (blue) at 24 hours in TREx FLAG-ORF11 OE cells.

(E). Protein disorder prediction for ORF11 using PrDOS software and FuzDrop Software. ORF11 protein sequence with amino acids removed for the truncation mutant in red and putative phosphorylated serine residues highlighted in the IDRs.

(F). IF staining of control TREx cells (Row 1), ORF11 CRISPR TREx cells (row 2), full length ORF11 rescue TREx (row 3), truncated ORF11 rescue (ΔIDR ORF11) (row 4) or serine mutated ORF11 (ΔSerine5 ORF11) (row 5). Staining is for SFPQ (red), DAPI (blue) and *NEAT1* (green). All cells were at 24 hours post-reactivation

(G). ORF11 protein purification and expression with coomassie Blue staining (left) and western blot probing for His-ORF11 for further confirmation of protein expression (right).

(H). FIDA analysis for ORF11 droplet forming propensity. Signal spikes are observed when ORF11 undergoes phase separation, with each spike signifying a droplet.

(I). IF of FUS KD HeLa cells or FUS KD HeLa cells overexpressing HA-ORF11. Stained for SFPQ (red), *NEAT1* (green) and DAPI (blue) (upper row). White arrows indicate co-localisation of SFPQ and *NEAT1*. IF of WT Hela or FUS KD HA-ORF11 Hela with staining for SFPQ (red), DAPI (blue) and either *NEAT1* or HA (green). White arrows highlight SFPQ and ORF11 co-localisation

(J). String analysis of TMT LC-MS/MS of FLAG (ORF11) Co-IPs in TREx FLAG-ORF11 O/E cells. (n=2) at 0 (left) and 24 hours (right). Inputs were analysed via western blot to confirm presence of FLAG-ORF11 and lytic replication. * denotes proteins of interest

(K). Table highlighting proteins of interest within the SFPQ and/or FLAG-ORF11 TMT-MS at 24 hours. Proteins are divided into DEAD/DEAH box helicases, hnRNPs or other known RNA processing factors.

(L-P) IF analysis of some of the TMT-MS enriched targets at 0 and 24 hours in TREx or TREx FLAG-ORF11 O/E cells with staining for **(L)** SFPQ (red) and DHX9 (green), **(M)** hnRNP M (red) and FLAG/ORF11 (green), **(N)**, DDX17 (red) and FLAG/ORF11 (green), **(O)** SFPQ (red) and DDX21 (green) **(P)** hnRNP U (red) and FLAG/ORF11 (green). All cells stained DAPI (blue). White arrows are used to highlight condensates.

Scale bars are 5 μm in length, except for zoom I where scale bar represents 2 μm.

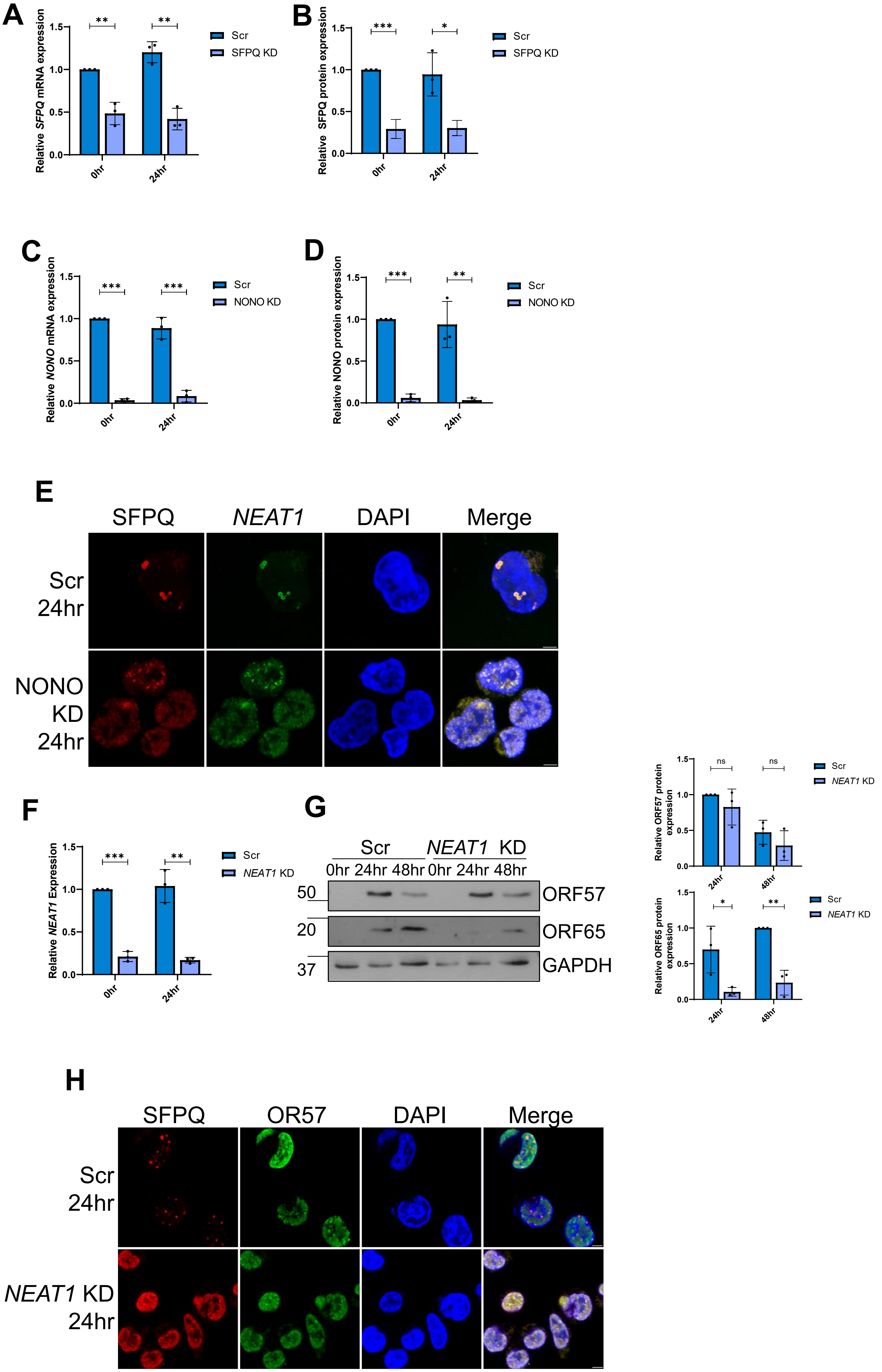
A
Relative SFPQ mRNA expression
Scr
SFPQ KD
0hr
24hr
B
Relative SFPQ protein expression
Scr
SFPQ KD
0hr
24hr
C
Relative NONO mRNA expression
Scr
NONO KD
0hr
24hr
D
Relative NONO protein expression
Scr
NONO KD
0hr
24hr
E
SFPQ
NEAT1
DAPI
Merge
Scr 24hr
NONO KD 24hr
F
Relative NEAT1 Expression
Scr
NEAT1 KD
0hr
24hr
G
Scr
NEAT1 KD
0hr 24hr 48hr 0hr 24hr 48hr
50
20
37
ORF57
ORF65
GAPDH
Relative ORF57 protein expression
Relative ORF65 protein expression
Scr
NEAT1 KD
24hr
48hr
H
SFPQ
OR57
DAPI
Merge
Scr 24hr
NEAT1 KD 24hr

Figure S4(A-H)

Figure S4.

(A). qPCR analysis of *SFPQ* levels at 0 and 24 hours in TREx scr and SFPQ KD cells. GAPDH was used as a housekeeper (n=3). P values are 0.0025 and 0.0016.

(B). Densitometry analysis of SFPQ protein levels in 0 and 24 hours in scr and SFPQ KD TREx cells (n=3). P values are 0.0004 and 0.0155.

(C). qPCR analysis of *NONO* levels in scr and NONO KD TREx cells at 0 and 24 hours. GAPDH was used as a housekeeper. (n=3). P values are <0.0001 and 0.0007.

(D). Densitometry analysis of NONO protein levels in 0 and 24 hours in scr and NONO KD TREx cells (n=3). P values are <0.0001 and 0.0048

(E). IF in scr or NONO KD TREx cells with staining against SFPQ (red), *NEAT1* (green) and DAPI (blue).

(F). qPCR analysis of *NEAT1* levels in scr or GapmeR treated TREx cells at 0 or 24 hours. GAPDH was used as a housekeeper, n=3. P values are <0.0001 and 0.0015.

(G). Representative western blot of ORF57 and ORF65 in scrambled and *NEAT1* GapmeR treated TREx at 0, 24 hours and 48 hours. GAPDH was used a loading control. Densitometry analysis on n=3. ORF57 P values are 0.3008 and 0.2945. ORF65 p values are 0.0367 and 0.0016.

(H). IF analysis of scrambled and *NEAT1* GapmeR treated TREx at 24 hours with staining for SFPQ (red), ORF57 (green) and DAPI (blue).

Scale bars 5 µm. All repeats are biological. In A-D, F-G data are presented as mean ± SD. *P < 0.05, **P < 0.01 and ***P < 0.001 (unpaired two-tailed Student’s t-test).

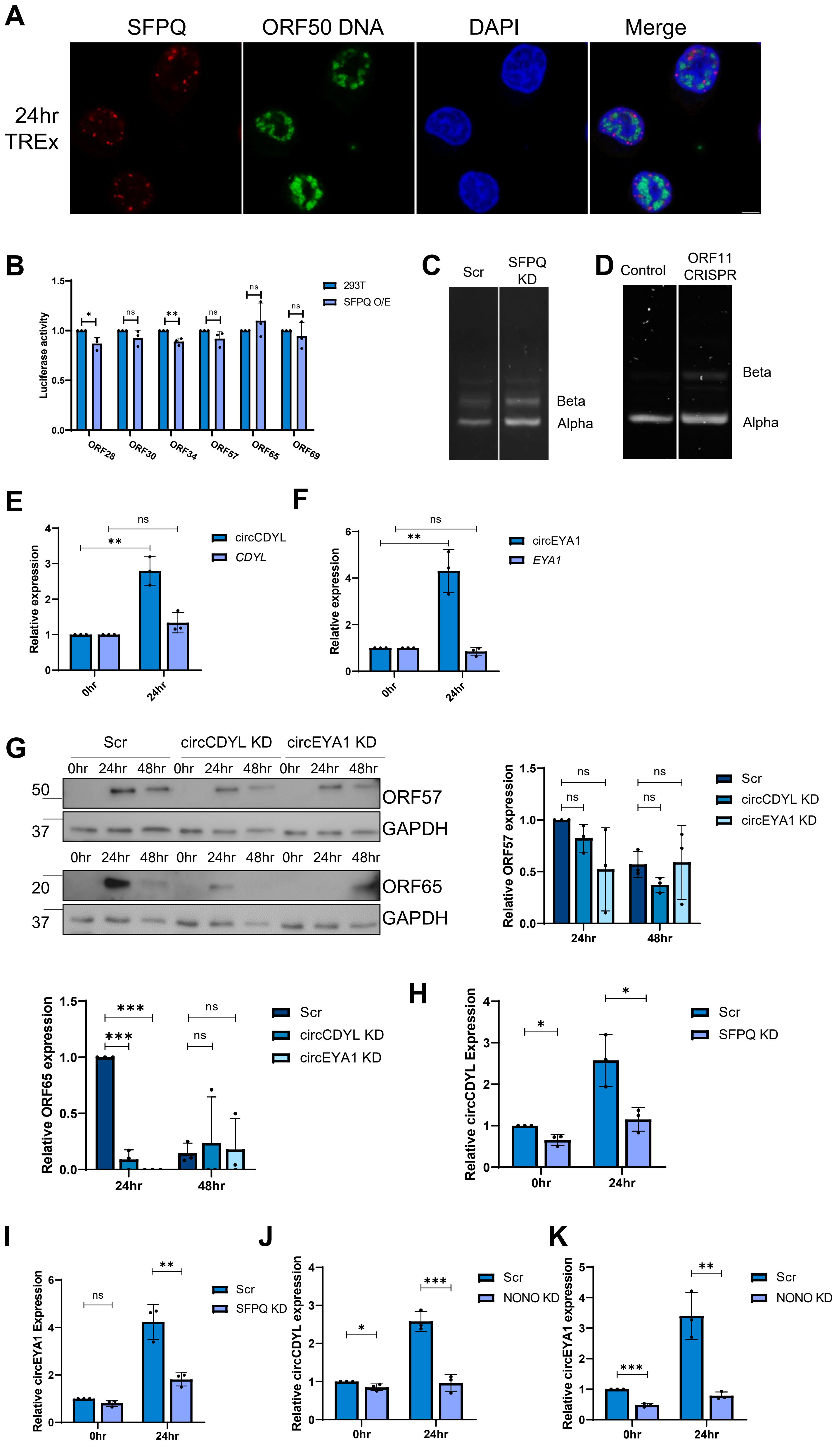
A
SFPQ
ORF50 DNA
DAPI
Merge
24hr TREx
B
Luciferase activity
293T
SFPQ O/E
ORF28
ORF30
ORF34
ORF57
ORF65
ORF69
C
Scr
SFPQ KD
Beta
Alpha
D
Control
ORF11 CRISPR
Beta
Alpha
E
Relative expression
circCDYL
CDYL
0hr
24hr
F
Relative expression
circEYA1
EYA1
0hr
24hr
G
Scr
circCDYL KD
circEYA1 KD
0hr
24hr
48hr
ORF57
GAPDH
ORF65
Relative ORF57 expression
Relative ORF65 expression
H
Relative circCDYL Expression
Scr
SFPQ KD
I
Relative circEYA1 Expression
Scr
SFPQ KD
J
Relative circCDYL expression
Scr
NONO KD
K
Relative circEYA1 expression
Scr
NONO KD

Figure S5(A-K)

Figure S5.

(A). IF in TREx cells at 24 hours with staining against SFPQ (red), ORF50 DNA (green) and DAPI (blue).

(B). Dual-luciferase reporter constructs containing viral 5' UTRs were transfected into HEK-293T and SFPQ-GFP OE cells and relative renilla luciferase activity was measured. The firefly luciferase activity was used as a transfection control, n=3. P values are 0.0226, 0.1773, 0.0062, 0.1417, 0.3830 and 0.5119.

(C-D). Representative Agarose gels for *K8* splicing assays for alpha and beta variant, for scr vs SFPQ KD TREx (C) and control vs ORF11 CRISPR cells (D).

(E). qPCR of circCDYL and *CDYL* levels at 0 and 24 hours in TREx, GAPDH was used as a housekeeper, n=3. P values are 0.0015 and 0.1125.

(F). qPCR of circEYA1 and *EYA1* levels at 0 and 24 hours in TREx cells, GAPDH was used as a housekeeper, n=3. P values are 0.0035 and 0.2198.

(G). Representative western blot of ORF57 and ORF65 levels in scrambled, circCDYL KD and circEYA1 KD TREx cells at 0, 24 and 48 hours. GAPDH was used as a loading control. Densitometry analysis was performed on n=3 for ORF57 and ORF65. P values are 0.6047, 0.0929, 0.4910 and 0.9914 for ORF57 and <0.00001, <0.0001, 0.8993 and 0.9844 for ORF65.

(H-I). qPCR of circCDYL and circEYA1 in scrambled and SFPQ KD TREx cells at 0 and 24 hours. GAPDH was used as a housekeeper. N=3. P values are 0.0100 and 0.0232 in H. P values are 0.0595 and 0.0059 in I.

(**J-K**). qPCR of circCDYL and circEYA1 in scrambled and NONO KD TREx cells at 0 and 24 hours. GAPDH was used as a housekeeper. N=3. P values are 0.0415 and 0.0012 in J. P values are 0.0001 and 0.0043 in K.

All repeats are biological. Scale bar represents 5 µm in A. In B, E-K data are presented as mean ± SD. *P < 0.05, **P < 0.01 and ***P < 0.001. Unpaired two-tailed Student's t-test was used for B, E-F, H and a one-way ANOVA was performed for G.

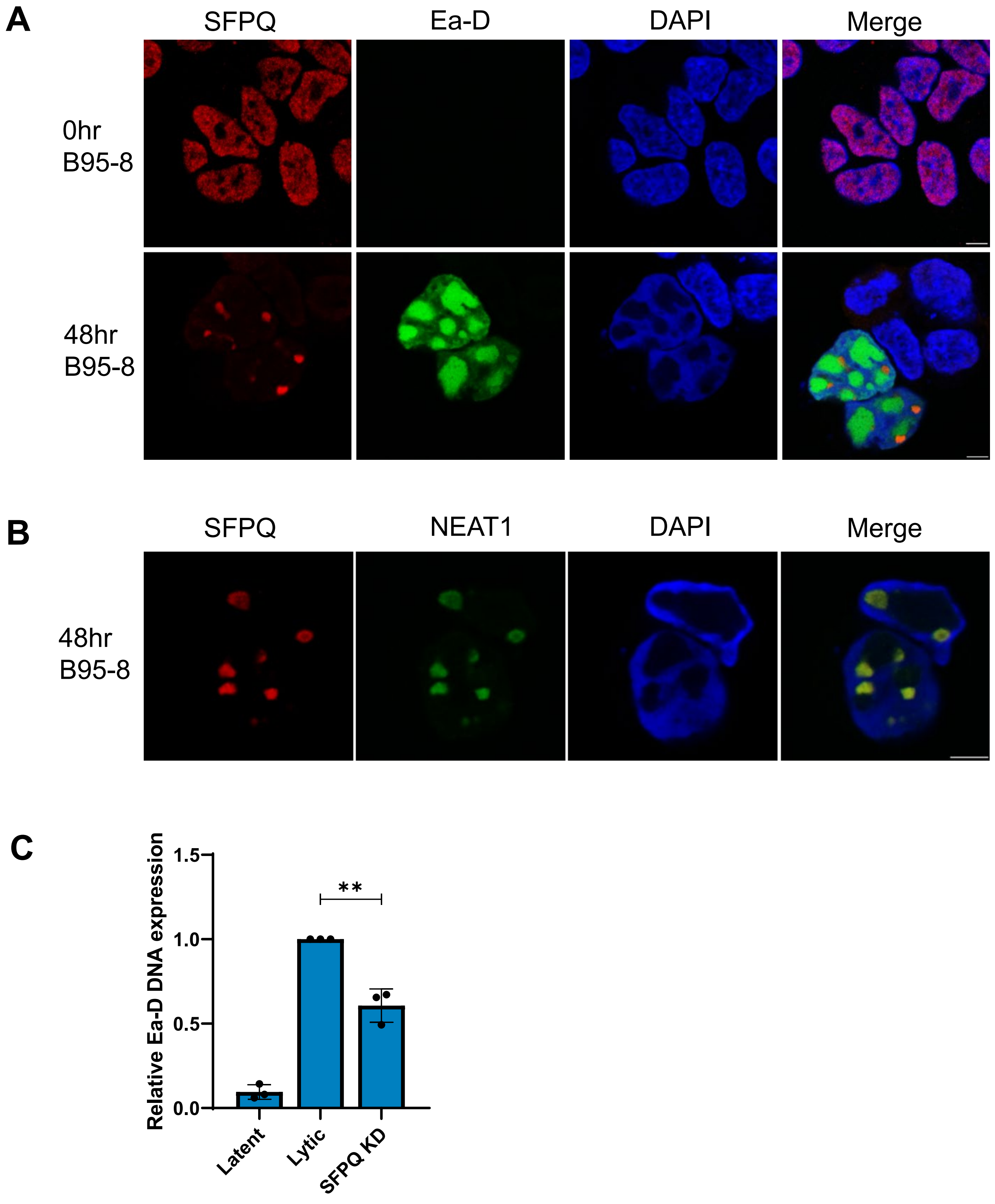

Figure S6.

(A). IF of 0 or 48 hour reactivated 293-SL-HB9-862-L1 Cells stained for SFPQ (red), Ea-D (green) and DAPI (blue).

(B). IF of 48 hour reactivated 293-SL-HB9-862-L1 Cells stained for SFPQ (red), *NEAT1* (green) and DAPI (blue).

(C). qPCR analysis of Ea-D DNA levels for viral load at 72 hr post-EBV induction, with scr and SFPQ KD 293-rJJ-L3 cells including uninduced cells as a reactivation control and GAPDH as a housekeeper (n = 3). P value is 0.0023.

In C data are presented as mean ± SD. *P < 0.05, **P < 0.01 and ***P < 0.001. Unpaired two-tailed Student's t-test was used for C. Scale bars are 5 μm.

Figure S6(A-C)

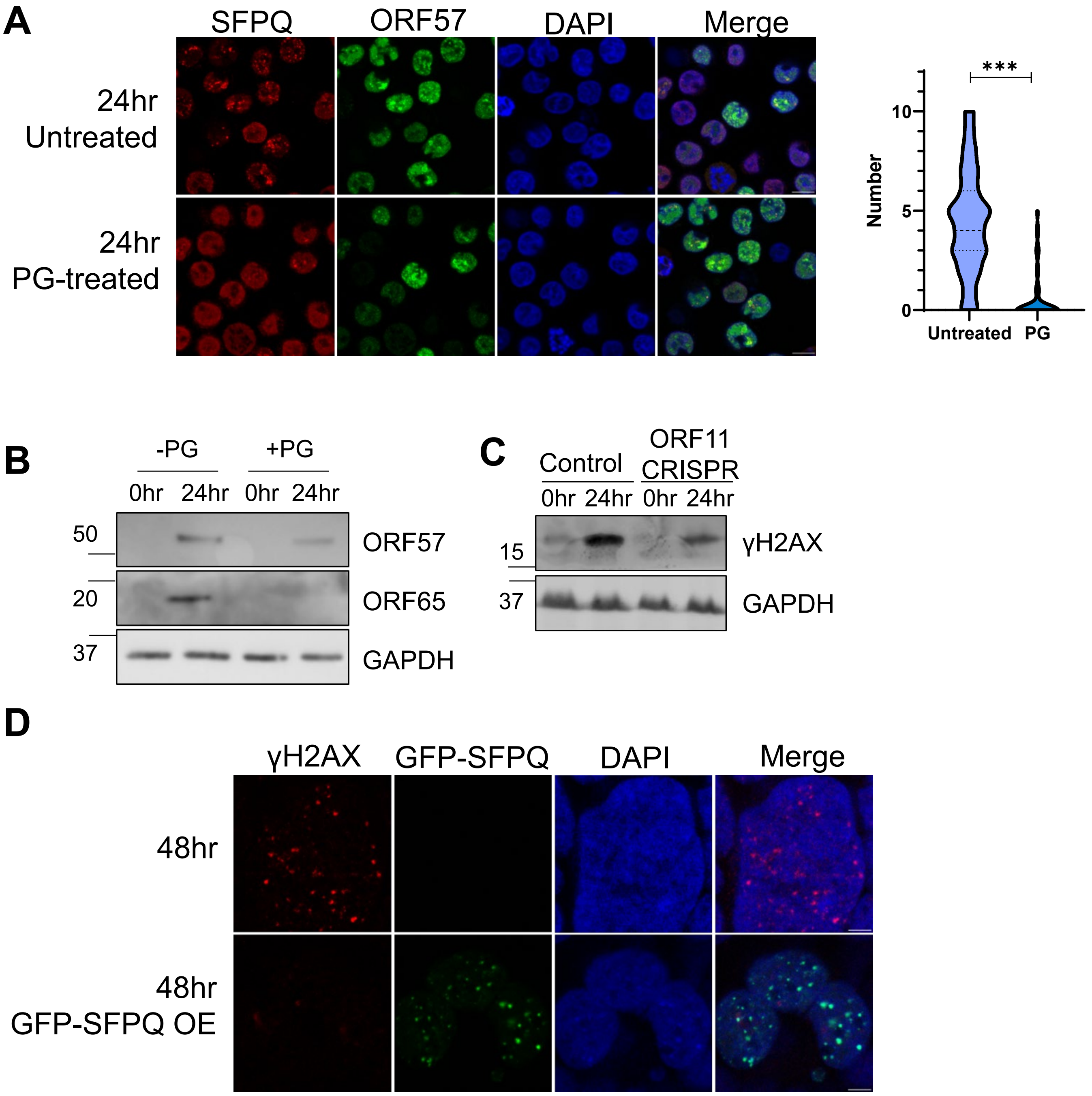

Figure S7.

(A). IF of untreated or 8 hour PG treated TREx cells. Cells were reactivated for 24 hours, with PG added at 16 hours post-lytic induction. Cells stained for SFPQ (red), ORF57 (green) and DAPI (blue). Number of v-mPS per cell were quantified in untreated and PG treated cells at 24 hours. 100 individual cells were counted per condition. P value is <0.0001.

(B). Representative western blot of ORF57 and ORF65 protein levels in PG treated TREx cells at 0 and 24 hours. GAPDH was used as a loading control, n=3.

(C). Representative western blot of γH2AX at 0 and 24 hours in control and ORF11 CRISPR cells. GAPDH was used as a loading control, n=3.

(D). IF of rJJ-L3-#1 at 48 hours either positive or negative for GFP-SFPQ OE. Cells were stained for γH2AX (red) and DAPI (blue).

Scale bars are 10 μm in A and 5 μm in D. Unpaired two-tailed Student's t-test was used for A. All repeats are biological.

Figure S7(A-D)

Primer	Forward	Reverse
**GAPDH**	TGTCAGTGGTGGACCTGA	GTGGTCCTTGAGGGCAATG
**ORF57**	GCCATAATCAAGCGTACTGG	GCAGAGAAATATTGCGGTGT
**K8**	AGGACCACACATTTCGCAAC	ACCCCTTGTCAGTTCTTC
**PAN**	ATAGGCGACAAAGTGAGGTGGCAT	TAACATTGAAAGAGCGCTCCCAGC
**ORF59**	GGTCCGGATATGCTCCTAGTT	CAGCATGCTCACGAGGAATA
**ORF8**	CTACTTCATCACCCGCAACG	TCCAGGTCAAACACGCTACT
**ORF9**	CGTTTACCACCGAGCTAAGC	TGTCGAAGTACAGGTCCACC
**ORF10**	GGACCTCCTGACATTCGAGT	AGGGGTTGTTATGAGGGGTG
**ORF65**	AAGGTGAGAGACCCCGTGAT	TCCAGGGTATTCATGCGAGC
**Ea-D**	CTAGCCGTCCTGTCCAAGTGC	AGCCAAACGCTCCTTGCCCA
**SFPQ**	ACAGGGAAAGGCATTGTTGA	TCATCTAGTTGTTCAAGTGGTTCC
**NONO**	TGATGAAGAGGGACTTCCAGA	AGCGCATGGCATATTCATACT
**NEAT1**	AGTACCCTGAGAGCCAGTATTGGT	GGCAGCTGAGTCAATCTCCTTT
**circPAN**	ACCAGACGGCAAGGTTTTA	TCGTTAGTCAACCTAGCAAAACA
**circvIRF4**	CTCCGTGTGGATACCAGTGA	TGGTCCCACGCAACAGTCT
**vIRF4**	CCCAACAGGCCAGCTACATAA	CTTCGTGGAACTCTGAGACGC
**EYA1**	GAGCTGATGGCTCCGAGTTT	GCTATGCGGGCTGGTTAGAT
**circEYA1**	TTGCTTACTGGGTCCTACGC	TACTGCTCCCAATTGCTGAA
**CDYL**	CGAGGAGCTGTACGAGGTTG	ACGAGTGCCTAAGGAGAGGT
**circCDYL**	ACCCACTAGTGCCTCAGGTG	CTCGCTGTCATGCCTTTCC
**circSPECC1**	GAGAGCTGCGAAGTTCAAGA	GCCTGTCCGTTTAGTTGTTGT
**ORF11**	CCCTAGCCACATCGTCATCT	TGCAGTGTAGGTCTCCGTTT
**ORF19**	CATAGACATGCCCCAAAGCC	TTCATGTACAGGGTGGGCAT
**ORF20**	CAACTCTCCGACTCGATCCA	ATTTTCCTTCGGTGGTGTGC
**ORF25**	CATATGCCACCCTCGAATGC	TGAGAGGTTGGGTGCAGTAG
**ORF58**	GCTGAATCTCGACCCCCATT	CTGACTCCGGTCCTGAATGG
**ORF61**	AATGAGCCGGGTACTTTGGA	CCAGAGTTCCATGTATGCGC
**ORF64**	TGAGGTCGGTTTTGAGTCGA	TATGACGTGTGCAGGACTGT
**ORF73**	GCAGACTACACCTCCACACT	GTAGATCGGGGACTCTGTGG
**K4**	CAGCGAGCAGTGACTGGTAA	GCATAGACCGGACAAGTGCT
**K10**	GGATACAGACGCGTGACAAC	CCAGCTCTGTCGGTAAATGC
**K8 alpha**	CCACCAAGAGGACCACACATTTC	CACACAAAGTCTGGCATGGTTCTCCC
**FUS**	AGCGGTGTTGGAACTTCG	GACTGCTCTGCTGGGAATAG
**ORF45**	TACCAGAGGAGGCGGTAGAC	TCCGGACGTGAACAATGAGG
**ORF69**	TCTGGGAGAAACTGCGCTTA	CAAGTGCGGTTCCCTTTTGA
**K7**	CTGCCGCTTCACCTATGGAT	ATTGCCAAAAGCGACGCAAT
**K8 beta**	CCACCAAGAGGACCACACATTTC	GCTGCCGGGATAACAATTCCTTC

Supplementary Table 1.

List of primers used in this study.

Supplementary Source data

2C

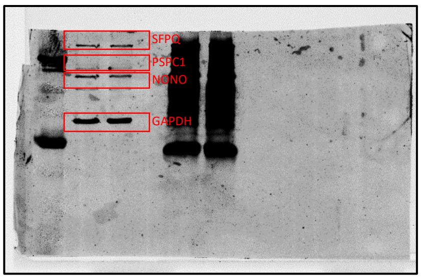

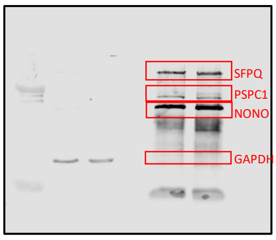

2E

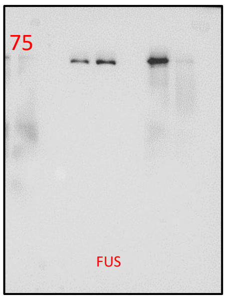

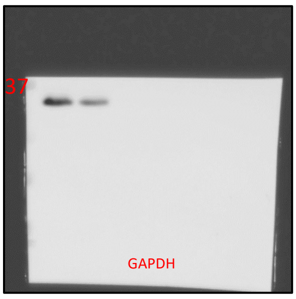

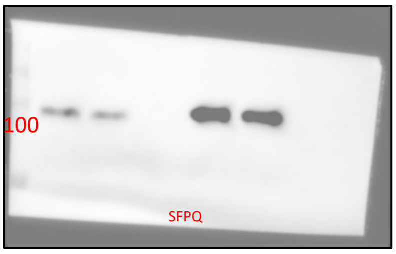

2F

	0hr			24hr		
Scr	1	1	1	1.261377	0.986233	0.864537
FUS KD	0.07911	0.077482	0.062935	0.061214	0.089622	0.079384

2G

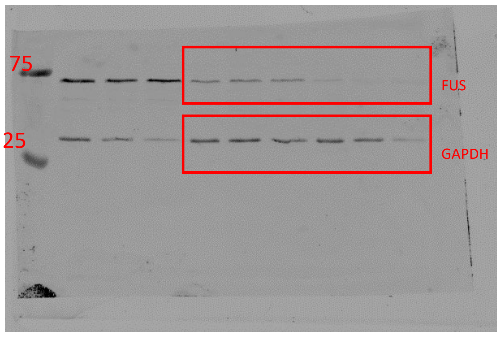

	Scr			FUS KD		
0	1	1	1	0.069592	0.020927	0.03442
24	1.420791	1.437153	1.300496	0.060206	0.026414	0.025284
48	1.005808	1.206363	1.096374	0.160881	0.051154	0.033442

2I

Scr	KD
1	0.222211
1	0.133972
1	0.085378

2J

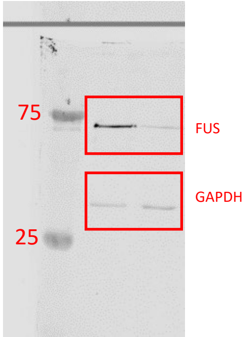

Scr	KD
1	0.118421
1	0.083562
1	0.080068

2P

16hr	24hr
10.9	100
21.7	85
20.8	88.88

3A

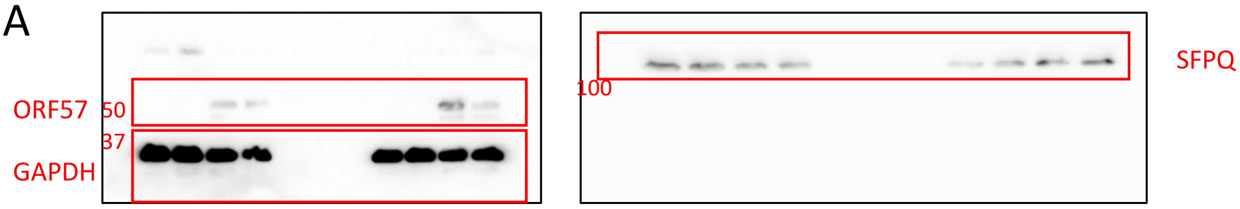
ORF57
50
37
GAPDH
100
SFPQ

3B

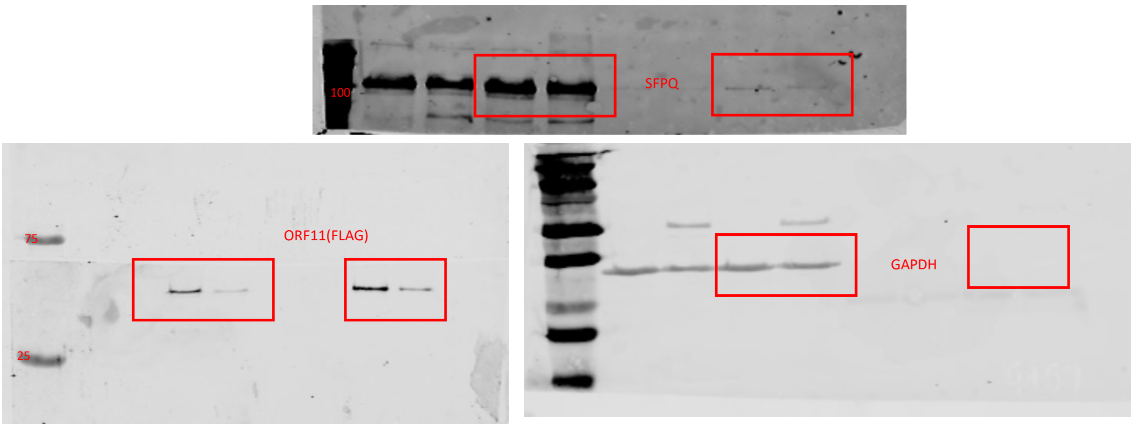
100
SFPQ
75
ORF11(FLAG)
25
GAPDH

3C

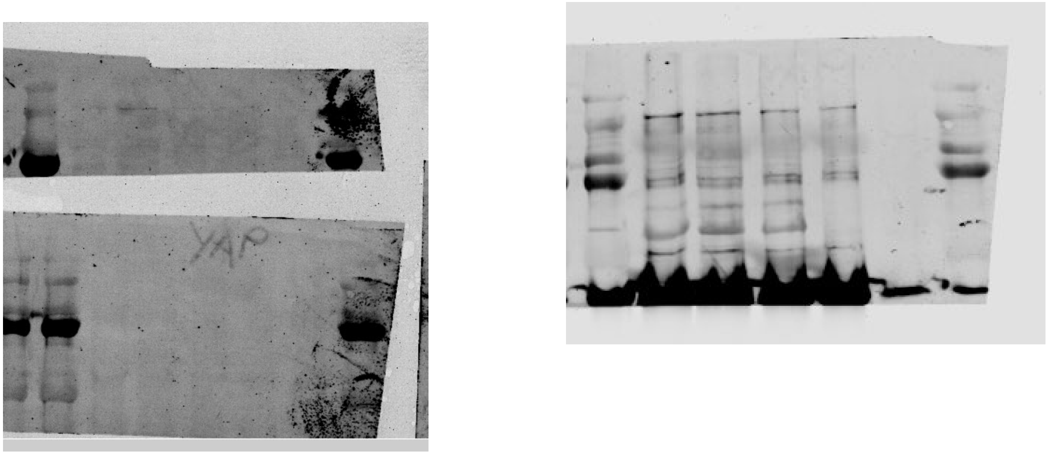

3G

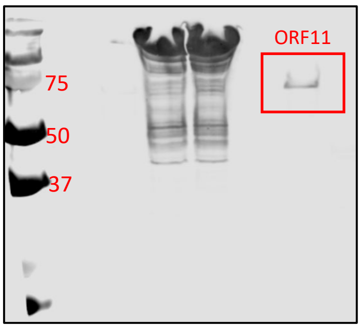
75
50
37
ORF11

3J

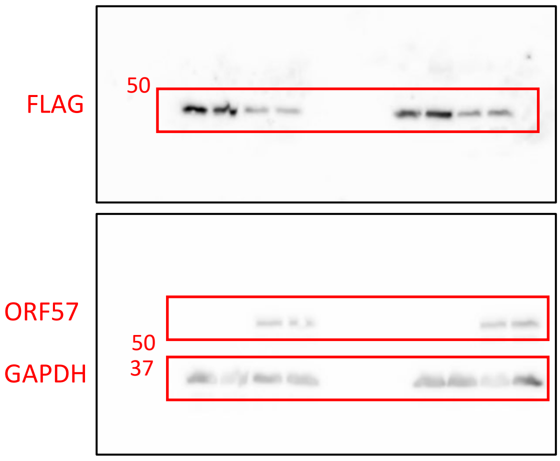
FLAG
50
ORF57
GAPDH
50
37

4A

	Scr			SFPQ KD		
0hr	1	1	1	0.598739	0.514057	0.340329
24hr	1.284524	1.060688	1.261377	0.565779	0.348686	0.342696

4B

	Scr			SFPQ KD		
0hr	1	1	1	0.42271	0.233725	0.219149
24hr	0.881241	1.228603	0.723191	0.406529	0.270741	0.232344

4C

	Scr			NONO KD		
0hr	1	1	1	0.042837	0.012958	0.051653
24hr	1.006956	0.752623	0.904379	0.148137	0.012132	0.091189

4D

	Scr			NONO KD		
0hr	1	1	1	0.067397	0.102457	0.009284
24hr	0.790856	0.766547	1.255449	0.060674	0.030187	0.008223

4F

	scr			NEAT1 KD		
0	1	1	1	0.156222	0.203298	0.27355
24	0.837987	1.22264	1.058154	0.130912	0.191003	0.184369

4G

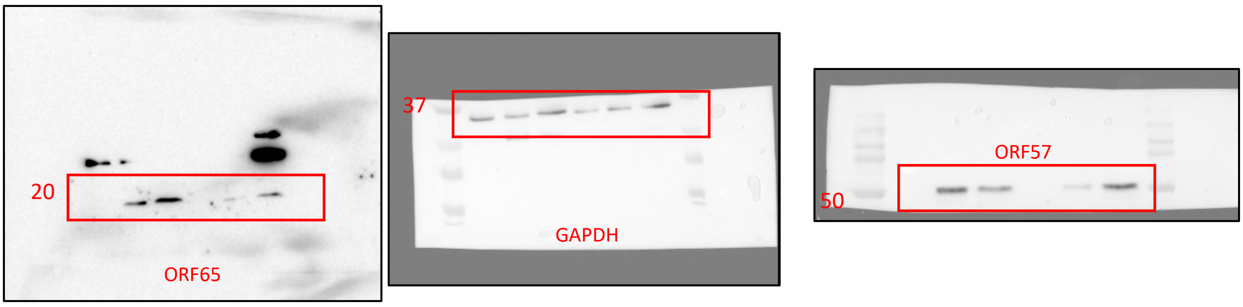

	Scr			NEAT1 KD		
24	1	1	1	0.909158	0.543209	1.027999
48	0.525538	0.286097	0.609374	0.203666	0.136013	0.524358

	Scr			NEAT1 KD		
24	0.761928	0.990124	0.345034	0.063163	0.174968	0.080454
48	1	1	1	0.344354	0.323595	0.033894

5B

	Scr 293T			SFPQ KD		
ORF28	1	1	1	0.92	0.89	0.8
ORF30	1	1	1	0.99	0.84	0.95
ORF34	1	1	1	0.9	0.92	0.85
ORF57	1	1	1	0.97	0.8315	0.956696
ORF65	1	1	1	1.29	0.94	1.07
ORF69	1	1	1	0.92	0.82	1.09

5C

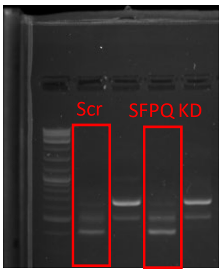

5D

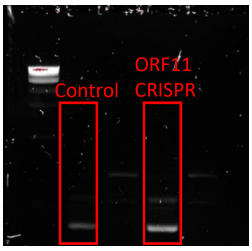

5E

	circCDYL			CDYL		
0hr	1	1	1	1	1	1
24hr	2.394957	2.789487	3.193194	1.156688	1.670176	1.185961

5F

	circEYA1			EYA1		
0hr	1	1	1	1	1	1
24hr	3.305801	4.438278	5.133704	0.993092	0.639493	0.904379

5G

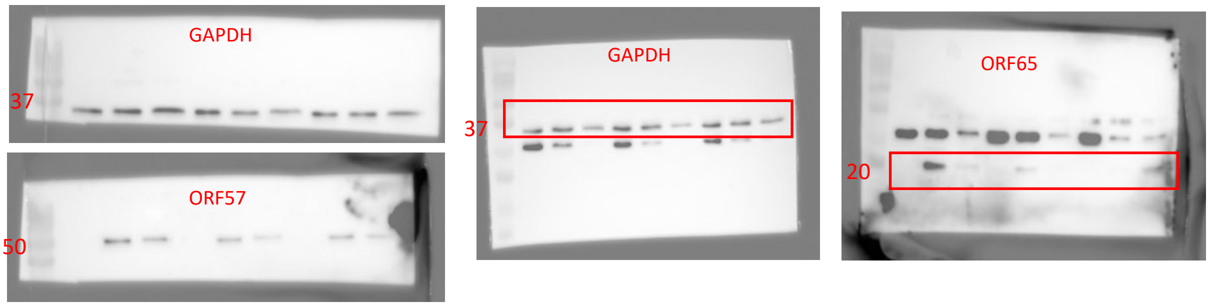

	ORF57								
	Scr			circCDYL KD			circEYA1 KD		
24	1	1	1	0.841778	0.680197	0.945792	0.559891	0.103456	0.905171
48	0.484014	0.714433	0.514525	0.435032	0.29375	0.391121	0.861161	0.18377	0.727336

	ORF65								
	Scr			circCDYL KD			circEYA1 KD		
24	1	1	1	0	0.17096	0.100048	0	0	0
48	0.104019	0.082157	0.249363	0.710035	0	0	0	4.98E-01	4.16E-02

5H

	Scr			SFPQ KD		
0hr	1	1	1	0.888843	0.864537	0.64842
24hr	3.386981	4.69134	4.626753	1.993081	1.94531	1.489677

5I

	Scr			SFPQ KD		
0hr	1	1	1	0.50698	0.737135	0.724471
24hr	1.93858	3.193194	2.592222	0.832199	1.252664	1.370783

5J

	Scr			NONO KD		
0hr	1	1	1	0.923382	0.752623	0.873573
24hr	2.877867	2.479415	2.386671	1.125058	1.046085	0.702222

5K

	Scr			NONO KD		
0hr	1	1	1	0.532185	0.420448	0.503478
24hr	3.271608	2.703822	4.213444	0.702222	0.732043	0.929805

6C

Latent	Lytic	KD
0.061214	1	0.655197
0.143587	1	0.671286
0.080772	1	0.493116

7A

Untreated	PG
0	0
0	0
0	0
0	0
0	0
0	0
0	0
0	0
1	0
1	0
1	0
1	0
1	0
2	0
2	0
2	0
2	0
2	0
2	0
2	0
2	0
2	0
2	0
3	0
3	0
3	0
3	0
3	0
3	0
3	0
3	0
3	0
3	0
3	0
3	0
3	0
3	0
3	0
3	0
4	0
4	0
4	0
4	0
4	0
4	0
4	0
4	0
4	0
4	0
4	0
4	0
5	0
5	0
5	0
5	0
5	0
5	0
5	0
5	0
5	0
5	0
5	0
5	0
5	0
5	0
5	0
5	0
5	0
5	0
5	0
5	0
6	0
6	0
6	0
6	0
6	0
6	0
6	0
7	0
7	0
7	0
7	1
7	1
7	1
7	1
7	1
8	1
8	2
8	2
8	2
8	3
8	3
9	3
9	3
9	3
9	4
10	4
10	4
10	5
10	5

7B

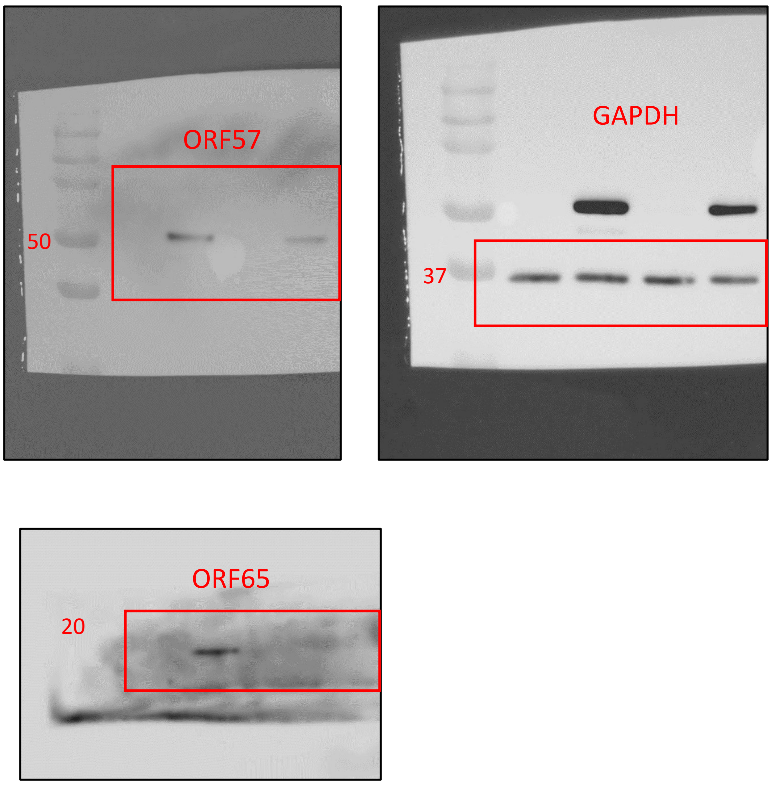

7C

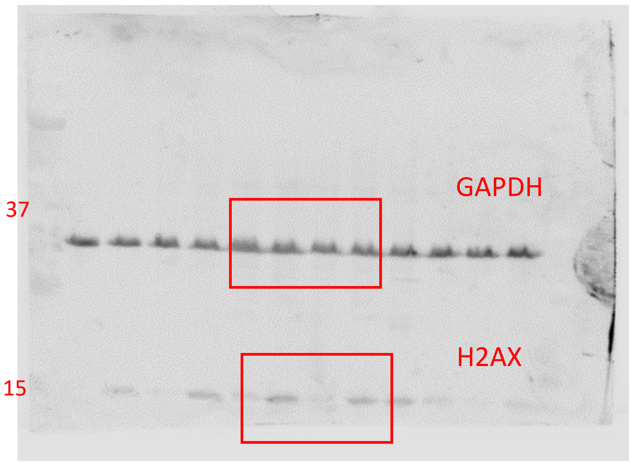

Supplement: Supplementary file 1 — Supplementary Information [file 41467_2024_54592_MOESM1_ESM.pdf]
