# Supplementary material for: Virus-modified paraspeckle-like condensates are hubs for viral RNA processing and their formation drives genomic instability
Source: Nat Commun. 2024 Nov 26;15:10240. doi: 10.1038/s41467-024-54592-5 (PMC11599752; doi:10.1038/s41467-024-54592-5)
Supplement: Supplementary file 3 — Description Of Additional Supplementary File [file 41467_2024_54592_MOESM3_ESM.pdf]

**Description of Additional supplementary files**

**Supplementary Movie 1**

Video of three-dimensional image acquisition (scale 1 um)

**Supplementary Movie 2**

Video of three-dimensional image acquisition (scale 0.7 um)
