# Supplementary material for: Virus-modified paraspeckle-like condensates are hubs for viral RNA processing and their formation drives genomic instability
Source: Nat Commun. 2024 Nov 26;15:10240. doi: 10.1038/s41467-024-54592-5 (PMC11599752; doi:10.1038/s41467-024-54592-5)
Supplement: Supplementary file 6 — Reporting summary [file 41467_2024_54592_MOESM6_ESM.pdf]

Corresponding author(s): Adrian Whitehouse

Last updated by author(s): Nov 5, 2024

Reporting Summary

Nature Portfolio wishes to improve the reproducibility of the work that we publish. This form provides structure for consistency and transparency in reporting. For further information on Nature Portfolio policies, see our Editorial Policies and the Editorial Policy Checklist.

Statistics

For all statistical analyses, confirm that the following items are present in the figure legend, table legend, main text, or Methods section.

n/a	Confirmed	
☐	☒	The exact sample size (*n*) for each experimental group/condition, given as a discrete number and unit of measurement
☐	☒	A statement on whether measurements were taken from distinct samples or whether the same sample was measured repeatedly
☐	☒	The statistical test(s) used AND whether they are one- or two-sided<br>*Only common tests should be described solely by name; describe more complex techniques in the Methods section.*
☐	☒	A description of all covariates tested
☐	☒	A description of any assumptions or corrections, such as tests of normality and adjustment for multiple comparisons
☐	☒	A full description of the statistical parameters including central tendency (e.g. means) or other basic estimates (e.g. regression coefficient) AND variation (e.g. standard deviation) or associated estimates of uncertainty (e.g. confidence intervals)
☐	☒	For null hypothesis testing, the test statistic (e.g. *F*, *t*, *r*) with confidence intervals, effect sizes, degrees of freedom and *P* value noted<br>*Give P values as exact values whenever suitable.*
☒	☐	For Bayesian analysis, information on the choice of priors and Markov chain Monte Carlo settings
☒	☐	For hierarchical and complex designs, identification of the appropriate level for tests and full reporting of outcomes
☒	☐	Estimates of effect sizes (e.g. Cohen's *d*, Pearson's *r*), indicating how they were calculated

Our web collection on statistics for biologists contains articles on many of the points above.

Software and code

Policy information about availability of computer code

Data collection	n/a
Data analysis	Graphpad prism 8, comet score 2.0, zen blue, rotorgene 6000, biorender, further RNA seq analysis in methods

For manuscripts utilizing custom algorithms or software that are central to the research but not yet described in published literature, software must be made available to editors and reviewers. We strongly encourage code deposition in a community repository (e.g. GitHub). See the Nature Portfolio guidelines for submitting code & software for further information.

Data

Policy information about availability of data

All manuscripts must include a data availability statement. This statement should provide the following information, where applicable:

- Accession codes, unique identifiers, or web links for publicly available datasets
- A description of any restrictions on data availability
- For clinical datasets or third party data, please ensure that the statement adheres to our policy

Quantitatve mass spectrometry datasets have been deposited to PRIDE (proteomics identifications database) and are available. RNA-Seq has been deposited to NCBI Geo. Details including links are in the methods sections.

Research involving human participants, their data, or biological material

Policy information about studies with human participants or human data. See also policy information about sex, gender (identity/presentation), and sexual orientation and race, ethnicity and racism.

Reporting on sex and gender	N/A
Reporting on race, ethnicity, or other socially relevant groupings	N/A
Population characteristics	N/A
Recruitment	N/A
Ethics oversight	N/A

Note that full information on the approval of the study protocol must also be provided in the manuscript.

Field-specific reporting

Please select the one below that is the best fit for your research. If you are not sure, read the appropriate sections before making your selection.

☒ Life sciences ☐ Behavioural & social sciences ☐ Ecological, evolutionary & environmental sciences

For a reference copy of the document with all sections, see nature.com/documents/nr-reporting-summary-flat.pdf

Life sciences study design

All studies must disclose on these points even when the disclosure is negative.

Sample size	No sample size calculation was conducted. We selected sample sizes of sufficient size to ensure reproducibility of our findings as well as large enough to perform stats analysis (at least n=3 unless stated). Our rational for selecting this sample size was primarily based on previous experience with these assays
Data exclusions	No data was excluded from this assay. We pre-established our exclusion criteria for failed replicates if either our positive or negative controls failed
Replication	We repeated experiments at least 3 times or number indicated in figure legends. Biological repeats
Randomization	Experiments were not randomised. Data variability controlled through inclusion of multiple biological replications and inclusion of multiple technical replicates within repeats when necessary.
Blinding	Researchers were not blinded

Reporting for specific materials, systems and methods

We require information from authors about some types of materials, experimental systems and methods used in many studies. Here, indicate whether each material, system or method listed is relevant to your study. If you are not sure if a list item applies to your research, read the appropriate section before selecting a response.

Materials & experimental systems

n/a	Involved in the study	
☐	☒	Antibodies
☐	☒	Eukaryotic cell lines
☒	☐	Palaeontology and archaeology
☒	☐	Animals and other organisms
☒	☐	Clinical data
☒	☐	Dual use research of concern
☒	☐	Plants

Methods

n/a	Involved in the study	
☒	☐	ChIP-seq
☒	☐	Flow cytometry
☒	☐	MRI-based neuroimaging

Antibodies

Antibodies used	Antibodies used are listed below: GAPDH (Proteintech 60004-1-Ig, WB 1/5000), ORF57 (Santa Cruz sc-135747, WB 1/1000, IF 1/100),

Antibodies used	ORF65 (CRB crb2005224, 1/100), SFPQ (Proteintech 15585-1-AP, WB 1/500, IF 1/50, RIP 1/50, IP 1/50), NONO (Proteintech 11058-1-AP, WB 1/1000, IF 1/100), PSPC1 (Proteintech 16714-1-AP, WB 1/3000, IF 1/50) FUS (Proteintech 11570-1-AP, WB 1/5000, IF 1/100), FLAG (Sigma F7425, WB 1/500), GFP (Proteintech 66002-1-Ig, WB 1/5000), Ku70 (Proteintech 10723-1-AP, WB 1/2000), γH2AX (CST, WB 1/1000, IF 1/100), SRSF2 (Novus Bio NB100-1774SS, IF 1/250), RNA pol II (Sigma-Aldrich 05-623, IF 1/50), SFPQ (Proteintech 67129-1-Ig, IF 1/400, IP 1/400), FLAG (Sigma F1804, IF 1/50), hnRNP U (Proteintech 14599-1-AP, IF 1/20), hnRNP M (Proteintech 26897-1-AP, IF 1/50), DDX17 (Proteintech 19910-1-AP, IF 1/10), DDX21 (Proteintech 66925-1-Ig, IF 1/50), DHX9 (Proteintech 67153-1-Ig, IF 1/50), HA (abcam ab9110, IF 1/50), Ea-D (Santa Cruz sc-58121, WB 1/500, IF 1/50, ICP27 (Santa Cruz sc-58121, IF 1/50), pp72 (Santa Cruz sc-69834, IF 1/50).
Validation	Each antibody was used as per commercial instructions and has previously been published in other studies

Eukaryotic cell lines

Policy information about cell lines and Sex and Gender in Research

Cell line source(s)	TREx-BCBL1-RTA cells were gift Jae. U Jung (Uni of Southern California). HEK-293T-rKSHV.219 were gift from Dr Jeffery Vieira (University of Washington). Human foreskin fibroblasts (HFFs) were a gift from J Sinclair (University of Cambridge). EBV cell lines were created by W. Wongwiwa. Hela and HEK-293Ts were purchased from ATCC.
Authentication	Cells came authenticated from ATCC. Virus containing cells routinely screened for virus,
Mycoplasma contamination	Cells tested neg for mycoplasma
Commonly misidentified lines (See ICLAC register)	HEK-293Ts. Cell line used for transfections to generate lentivirus

Plants

Seed stocks	*Report on the source of all seed stocks or other plant material used. If applicable, state the seed stock centre and catalogue number. If plant specimens were collected from the field, describe the collection location, date and sampling procedures.*
Novel plant genotypes	*Describe the methods by which all novel plant genotypes were produced. This includes those generated by transgenic approaches, gene editing, chemical/radiation-based mutagenesis and hybridization. For transgenic lines, describe the transformation method, the number of independent lines analyzed and the generation upon which experiments were performed. For gene-edited lines, describe the editor used, the endogenous sequence targeted for editing, the targeting guide RNA sequence (if applicable) and how the editor was applied.*
Authentication	*Describe any authentication procedures for each seed stock used or novel genotype generated. Describe any experiments used to assess the effect of a mutation and, where applicable, how potential secondary effects (e.g. second site T-DNA insertions, mosiacism, off-target gene editing) were examined.*
